# MicroRNA-21 Knockout Improve the Survival Rate in DSS Induced Fatal Colitis through Protecting against Inflammation and Tissue Injury

**DOI:** 10.1371/journal.pone.0066814

**Published:** 2013-06-24

**Authors:** Chenzhang Shi, Yong Liang, Jun Yang, Yang Xia, Hongqi Chen, Huazhong Han, Yongzhi Yang, Wen Wu, Renyuan Gao, Huanlong Qin

**Affiliations:** Department of General Surgery, Affiliated Sixth People’s Hospital, Shanghai Jiao Tong University School of Medicine, Shanghai Jiao Tong University, Shanghai, China; Charité, Campus Benjamin Franklin, Germany

## Abstract

**Background:**

MicroRNA-21 (miR-21) is overexpressed in most inflammatory diseases, but its physiological role in gut inflammation and tissue injury is poorly understood. The goal of this work is to understand the role of miR-21 in colitis and damage progression of intestine in a genetically modified murine model.

**Methods:**

Experimental colitis was induced in miR-21 KO and wild-type (WT) mice by 3.5% dextran sulphate sodium (DSS) administration for 7 days. Disease activity index(DAI), blood parameters, intestinal permeability, histopathologic injury, cytokine and chemokine production, and epithelial cells apoptosis were examined in colons of miR-21 KO and WT mice.

**Results:**

miR-21 was overexpressed in intestine of inflammatory bowel diseases (IBD) and acute intestinal obstruction (AIO) patients when compared with normal intestinal tissues. Likewise, miR-21 was up-regulated in colon of IL-10 KO mice when compared with control mice. WT mice rapidly lost weight and were moribund 5 days after treatment with 3.5% DSS, while miR-21 KO mice survived for at least 6 days. Elevated leukocytes and more severe histopathology were observed in WT mice when compared with miR-21 KO mice. Elevated levels of TNF-α and macrophage inflammatory protein-2(MIP-2) in colon culture supernatants from WT mice exhibited significant higher than miR-21 KO mice. Furthermore, CD3 and CD68 positive cells, intestinal permeability and apoptosis of epithelial cells were significantly increased in WT mice when compared with miR-21 KO mice. Finally, we found that miR-21 regulated the intestinal barrier function through modulating the expression of RhoB and CDC42.

**Conclusion:**

Our results suggest that miR-21 is overexpressed in intestinal inflammation and tissue injury, while knockout of miR-21 in mice improve the survival rate in DSS-induced fatal colitis through protecting against inflammation and tissue injury. Therefore, attenuated expression of miR-21 in gut may prevent the onset or progression of inflammatory bowel disease in patients.

## Introduction

MicroRNAs (miRNAs) belong to a class of small RNA molecules that regulate gene expression at the post transcriptional level. MicroRNA-21 (miR-21) is a unique miRNA that it is overexpressed in most inflammatory diseases, tumour, and cardiac disease.[1]–[5] miRNA profiling have implicated it in inflammatory bowel disease (IBD).[6]–[8] Meanwhile, multiple global gene expression profiles have demonstrated that IBD is associated with differential expression of genes including inflammatory mediators, antimicrobial factors, and cell cycle regulators that are involved in inflammation and tissue remodeling.[9]–[11] Therefore, miR-21 is likely involved in the pathogenesis of IBD via regulation of relevant gene expression. It may play an important role in normal immune response and have altered expression in multiple immune-mediated disorders in IBD.

Increasing evidences have indicated that miR-21 play an important role in regulating inflammatory processes. It has been shown that miR-21 was primarily detected in the cytoplasm of mononuclear and multinucleated myeloid cells and its expression significantly increases in asthma. [12] Moreover, it plays an important role in maintaining effector phase of the T cells [13]. miR-21 and miR-34a mediated inhibition of endogenous WNT1 and JAG1 expression was important for proper monocyte derived dendritic cell (MDDC) differentiation. [14] It suppressed IL-12 production by targeting IL-12p35, which impaired anti-mycobacterial T cell responses both in vitro and in vivo. Additionally, miR-21 also promoted dendritic cell (DC) apoptosis by targeting Bcl-2. [15].

MicroRNA-21 is involved in diverse biological processes, including cell differentiation, proliferation, and apoptosis, presumably through its various targets. miR-21 was first noted as an apoptotic suppressor in various cell lines [16]. The anti-apoptotic function of AKT is partly through miR-21-dependent suppression of Fas ligand. [17] miR-21 expression was up-regulated during early stages of liver regeneration and targeting of *Peli1* provides a negative feedback loop regulating NF-κB signaling. [18] Knocking down of miR-21 impaired cell cycle progression of hepatocytes into S phase, mainly through a decrease in cyclin D1 protein level. [19] miR-21 is negatively regulated in G1-S transition, and participates in DNA damage-induced G2-M checkpoint through down-regulation of Cdc25A. [20] Moreover, miR-21 inhibited the metabolism of superoxide to hydrogen peroxide by directly attenuating SOD3. Alternatively, miR21 can reduce SOD2 level by inhibition of TNF-α production. [21].

Although multiple targets of miR-21 have been identified, including PTEN, PDCD4, FasL, SOD3, Cdc25A, RhoB, IL-12p35, Bcl-2, Pellino, TPM1, JAG1 and WNT1[4], [5], [12], [14], [15], [17]–[21], the role of miR-21 in gut damage and inflammation remains unclear. To explore the role of miR-21 in gut damage and inflammatory response *in vivo*, we developed miR-21 knockout (miR-21 KO) mouse model. Taking advantage of this unique genetic model together with dextran sodium sulphate (DSS)-induced experimental colitis, we examined the hypothesis that the colonic epithelial damage and cell apoptosis in colitis may be linked to the differential expression of miR-21.

## Materials and Methods

### Mice

Homozygous IL-10 knockout (IL-10 KO) and wild-type (WT) 129/SvEv mice (Jackson Laboratory, Bar Harbor, ME) were used in the study. Colon tissues were obtained from the IL-10 KO and WT controls mice at age of 16 weeks. miR-21 KO mice(Obtained from Prof Zonglai Jiang, School of Life Sciences & Biotechnology, Shanghai Jiao Tong University, Shanghai, China) and wild-type (WT) C57BL/6J controls were housed in specific pathogen-free conditions with free access to food and water.

### Genotype Analysis

**Figure 1:**
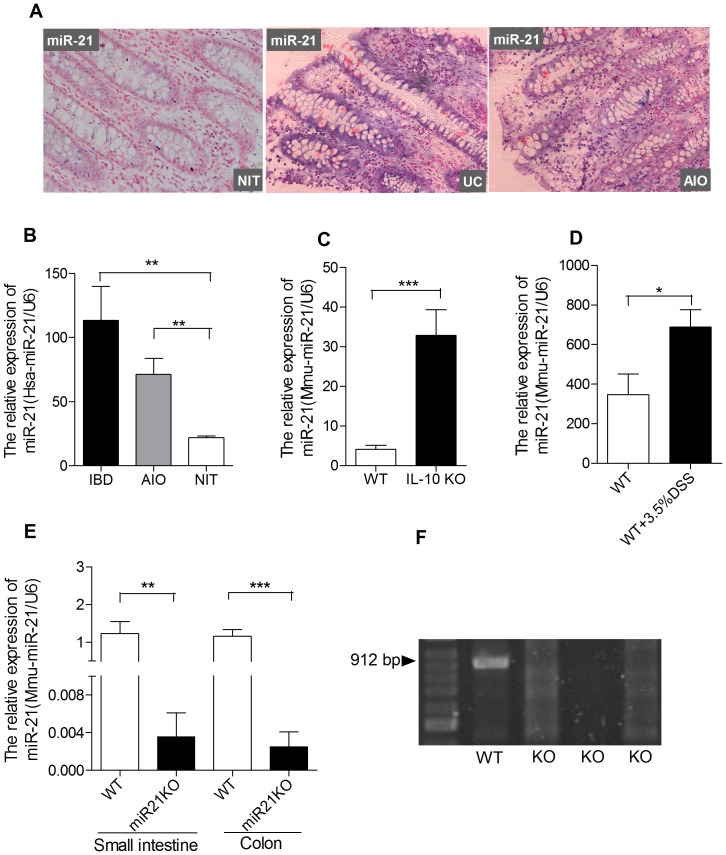
Expression of miR-21 in inflammation- and injury-involved intestine and miR-21 KO mice. (A) Representative photomicrographs in situ detection (200×magnification, n = 3 per group) and (B) QRT-PCR (n = 5 per group) shown miR-21 is overexpressed in intestinal tissue of IBD and AIO patients when compared with normal intestinal tissues (NIT) of human. (C) miR-21 is overexpressed in inflammatory colon tissue of IL-10 KO mice (n = 8) compared with WT mice (n = 8). (D) The expression of miR-21 in colon is significantly increased in DSS-treated WT mice compare to control mice (n = 5 per group). (E) Loss of expression of miR-21 in small intestine and colon of miR-21 KO mice (n = 5) compared with WT mice (n = 5). (F) PCR genotyping of miR-21 KO mice DNAs. (mean±SEM, *p<0.05, **p<0.01,***p<0.001; Student’s t test).

Mice were genotyped using PCR analysis of tail genomic DNA. Mouse sequence-specific primers for discerning between wild-type and miR-21 KO were as follows: Forward 5′-AACATAAAC CAACCAGCCAAC, Reverse 5′-CTTTGATCATTCCGAAATTGT. 32 cycles were set at 95°C, 60°C and 72°C each for 60 seconds respectively. A 912 bp amplicon defined wild-type and no amplification product reflected the miR-21 KO (Figure 1D).

### Patients

Intestinal tissues were obtained from surgical specimens of patients with IBD, acute intestinal obstruction (AIO) and as control from normal areas of the intestine of patients admitted for bowel resection because of polyps or diverticulosis. Specimens were frozen in optimum cutting temperature on ice and stored at −80°C refrigerator. Human studies were approved by the ethical committee of the affiliated Sixth People’s Hospital of Shanghai Jiao Tong University. We have written informed consent and confirmation were obtained from the patients.

### In situ Hybridization

In situ hybridization was performed with 5′-locked digoxigenin- labeled LNA™ miR-21 probe complementary to human mature miR-21 and LNA™ U6 snRNA as positive control (Exiqon, Vedbaek, Denmark). [22], [23] Briefly, human tissues were deparaffinized and deproteinized with protease K for 15 minutes at 37°C. Slides were then washed twice in PBS and dehydrate with ethanol. Hybridization was performed at 37°C for 28 hours, followed by blocking with 0.3% BSA in PBS for 30 minutes. The probe–target complex was detected immunologically by incubating with a digoxigenin Ab conjugated to alkaline phosphatase acting on the chromogen NBT/5-bromo-4-chloro-3-indolyl phosphate (Sigma) for 16 hours. Slides were counterstained with nuclear fast red, examined and photographed (Nikon Eclipse 80i, Japan).

### DSS-induced Experimental Colitis

To induce experimental colitis, 12–14 weeks mice were permitted free access to 3.5% DSS (w/v, MW = 36,000–50,000; MP Biomedicals) in drinking water for up to 7 days in acute colitis experiments. Fresh DSS solution was provided every day. The mice were checked each day for morbidity and weight was recorded. Mice were sacrificed by cervical dislocation at day 7 or judged as moribund before day 7 and immediately sacrificed, and the colons were removed, length and weight were measured. Induction of colitis was determined by weight loss, fecal blood, and diarrhea. [24] Animal studies were approved by the ethical committee of the affiliated Sixth People’s Hospital of Shanghai Jiao Tong University.

### Scoring of Disease Activity Index

Clinical disease activity index (DAI) ranging from 0 to 4 was calculated using the following parameters: stool consistency (normal, loose, diarrhea), presence or absence of fecal blood (guaiac paper test and macroscopic evaluation of the anus), and weight loss. Scoring of DAI was as described [24]–[28] in a blinded fashion.

### Haematological Analysis

100 µl of blood was collected from the tip of the tail vein at 4 day of 3.5% DSS treatment. Blood was drawn into EDTA-containing tubes (Axygen, USA). After vigorous mixing, complete blood count determined in an Sysmex xs-800i blood analysis system (Sysmex Corporation, Japan) using mouse specific settings. White blood cell (WBC) population was determined by blood smear, and corrected by WBC count.

### Intestinal Permeability in vivo

This measurement is based on the intestinal permeability towards fluorescein isothiocyanate-dextran (FITC-Dextran) (average mol wt 4,000 kDa, Sigma-Aldrich, USA) as described [29]–[31]. _ENREF_7_ENREF_7_ENREF_7_ENREF_7Briefly, mice that had fasted for 6 h given FITC-Dextran by gavage (0.6 mg/g body weight, 50 mg/ml). After 1 h, 120 µl of blood was collected from the tip of the tail vein in mice before DSS treatment. We collected 120 µl blood by cardiac puncture after given FITC-Dextran 1 h when end of the experiment or time of dying. The blood was centrifuged at 4°C, 10 000 g for 5 min. Plasma was diluted in an equal volume of PBS (pH 7.4) and analyses for FITC-Dextran concentration with a Synergy HT Multi-Mode Microplate Reader (BioTek, USA) at an excitation wavelength of 485 nm and emission wavelength of 528 nm. Standard curves were obtained by diluting FITC–dextran in non-treated plasma diluted with PBS (1∶3 v/v).

### Cytokine and Chemokine Production by Colon Culture

Colon tissue from rectum to caecum (10 mm) was washed in cold phosphate-buffered saline (PBS) supplemented with penicillin, streptomycin and amphotericin B (Sangon, Shanghai). Dissected segments were cultured in 12-well, flat-bottom culture plates (Corning Incorporated, NY, USA) in serum-free RPMI 1640 medium with antibiotics. After 24 h at 37°C, samples were centrifuged and supernatants stored at −80°C. Macrophage inflammatory protein 2 (MIP-2) and TNF-α were determined by ELISA kits (EIAab Science Co.,Ltd, Wuhan, China). In some experiments, serum was obtained by centrifugation, stored at −80°C, and then MIP-2 and TNF-α was measured.

### Quantitative Real-time Polymerase Chain Reaction (QRT-PCR) Analysis

Total RNA from colonic mucosa were prepared using Total RNA Extraction Kit (SLNco, Cinoasia, China). First strand synthesis of cDNA was performed with the Reverse Transcription Kit (TOYOBO, Japan). For analysis of miR-21, CDC42, PDCD4, NF-κB, CDC25A Cyclin D1 and RhoB, primers were used as listed (Table 1). 40 cycles were carried out for quantitative PCR with 95°C, 60°C and 72°C for 60 seconds each. Reactions were run on the FTC-3000 Qrt-PCR machine (Funglyn, Canada).

**Table 1:** Primers Used for Quantitative Real-time PCR.

Gene name	Primer sequence (5′ to3′)	amplicon size
m-GAPDH-F	AGGTTGTCTCCTGCGACTTCA	143bp
m-GAPDH-R	GAGGTCCACCACTCTGTTGCT	
m-Cdc42-F	CGACCGCTAAGTTATCCACAG	278bp
m-Cdc42-R	AGGGCAGAGCACTCCACAT	
m-Cdc25A-F	AGACCACGACACCTTTCACCTC	284bp
m-Cdc25A-R	CATTCTTCATATTCTCGCCATCC	
m-CyclinD1-F	CGCCCTCCGTATCTTACTTCA	108bp
m-CyclinD1-R	CTTCGCACTTCTGCTCCTCAC	
m-NF-κB-F	GTGCCAAGAGTGATGACGAGG	111bp
m- NF-κB -R	ATGCCAAGGCGATGGGTTC	
m-PDCD4-F	AACTATGATGACGACCAGGAGAAC	200bp
m-PDCD4-R	GCTAAGGACACTGCCAACACC	
m-RhoB-F	GACGGCAAGCAGGTGGAG	191
m-RhoB-R	ATGGGCACATTGGGGCAG	
U6-F	CTCGCTTCGGCAGCACA	88bp
U6-R	AACGCTTCACGAATTTGCGT	
miR-21-F	ACACTCCAGCTGGGTAGCTTATCAGACTGATG	70bp
miR-21-R	TGTCGTGGAGTCGGCAATTC	

F, Forward; R, Reverse.

### Histopathological and Immunofluorescence Analyses of Mouse Colon Tissue

Colon segments were fixed in 10% neutral-buffered formalin, embedded in paraffin, sectioned at 5 µm, and stained with H&E for histopathological detection of severity of inflammation, extent of injury and crypt damage. Scoring of histopathology was done as described [27], [28], [32] by an individual blinded to the treatment.

Formalin-fixed, paraffin-embedded colon sections were deparaffinized in xylene and rehydrated with alcohol. Samples were boiled for 10 min in antigen retrieval solution (SLNco, CinoAsia, China) and left at room temperature for 30 min. Slides were then incubated (1h, room temperature) with primary antibodies for CD3 (1∶100, Epitomics, Callifornia, USA), CD68 (1∶50, Abcam, UK). After three times washing with PBS, the slides were incubated for 45 min, room temperature with FITC labeled secondary antibodies (Jackson Immuno, Darmstadt, Germany). Slides were microscopic examined and photographed (Nikon Eclipse 80i, Japan).

### TUNEL Assay

TUNEL (deoxynucleotidyl transferase–mediated deoxyuridine triphosphate) (Beyotime, China) assay using paraffin-embedded tissues was performed as manufacturer’s instructions. Slides were observed under fluorescent microscopy (Nikon Eclipse 80i, Japan).

### Statistical Analysis

Data were analyzed by GraphPad Prism 5 software (San Diego, CA) and expressed as mean±SEM. Survival experiments were shown as KaplaneMeier plots and differences evaluated by two-tailed log-rank test. Differences in parametric data were evaluated by the Student’s two-tailed t test. Differences with p<0.05 were considered statistically significant.

## Results

### Up-regulation of miR-21 in Inflammation- and Injury-involved Intestine

To investigate the expression levels of miR-21 in different pathological conditions in intestine, we performed in situ hybridization of miR-21 in 3 ulcerative colitis (UC), 3 acute intestinal obstruction (AIO), and 3 control colonic specimens. (Figure 1A). Meanwhile, we performed QRT-PCR of 5 histologically control and 5 AIO- and 5 IBD-involved mucosa derived from surgical specimens. (Figure 1B) Moreover, we performed QRT-PCR in colon of 8 WT and 8 IL-10 KO mice (Figure 1C). Normal colonic mucosa of human show low expression of miR-21 when compare with AIO- and IBD-involved mucosa (Figure 1A,B). Meanwhile, the study of Wu et al. has found that miR-21 is up-regulation in active UC compare to inactive UC. [6] Consistently, miR-21 is significant overexpressed in colon of IL-10 KO mice when compare with control mice (Figure 1C). These results are consistent with the study of Schaefer et al that the expression of miR-21 is up-regulated in development of colonic inflammation in IL-10 KO mice. [33] In addition, the expression of miR-21 is significantly increased in DSS-treated colon of WT mice when compare with untreated WT mice (Figure 1D).

### Decreased Susceptibility to Experimental Colitis in miR-21 KO Mice

**Figure 2:**
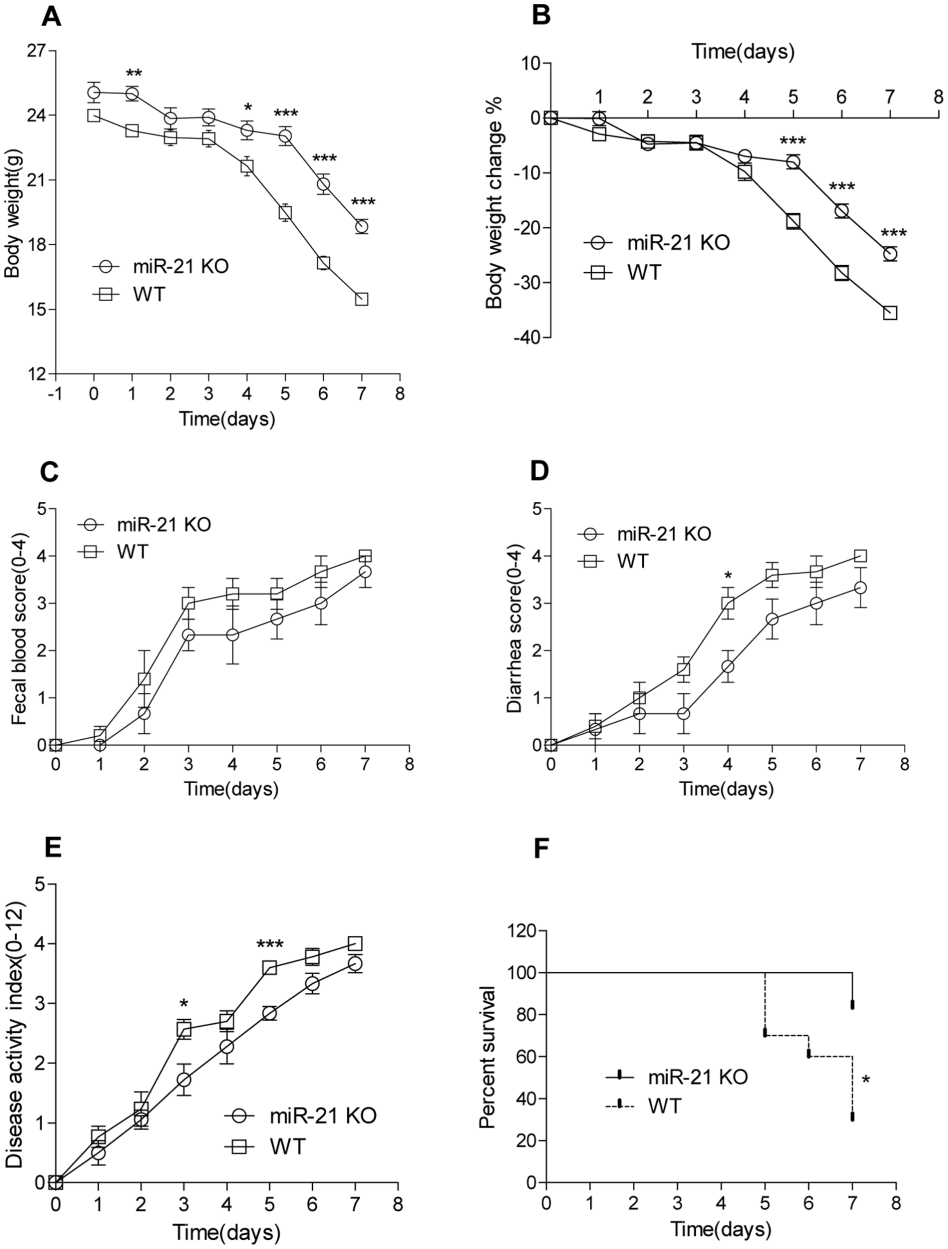
Severe experimentally induced colitis in miR-21 KO mice. MiR-21 KO and WT mice in C57BL/6J background were continuously administered 3.5% dextran sodium sulphate (DSS) freely available in drinking water. (A) Changes in body weights in WT (n = 10) and miR-21 KO (n = 6) mice were measured. (*p<0.05, **p<0.01, ***p<0.001; Student’s t test). Disease severity was measured daily and expressed in terms of (B) body weight loss,(C) fecal blood, (D) diarrhea, and (E) disease activity index (n = 6 per time point for miR-21 KO mice compared with WT control n = 10, *p<0.05, ***p<0.001, Student’s t test). (F) Percentage survival (n = 6 for miR-21 KO mice and n = 10 for WT mice, *p<0.05, log-rank test).

Mice carrying miR-21 KO allele offers us a unique tool to examine its role in intestine. To determine the knockout efficiency, we performed QRT-PCR to confirm loss of miR-21 expression in small intestine and colon of miR-21 KO mice (Figure 1E). Homozygous miR-21 KO mice in C57BL/6 background were intercrossed to generate miR-21 KO mice (Figure 1F). To investigate the role of miR21 in colitis, experimental colitis was induced by the administration of 3.5% DSS in drinking water for up to 7 days. WT mice lost weight more rapidly than miR-21 KO mice, with significant difference in body weight at day 5. The greatest percentage of body weight lost in WT and miR-21 KO mice were 36.5% and 29.1%, respectively.(Figure 2A, B) Meanwhile, WT mice are earlier appearance of fecal blood (on day 2) and diarrhea (on day 3) than miR-21 KO mice (Figure 2C, D). MiR-21 KO mice have a significant amelioration in the severity of DSS-colitis at day 5, as shown by a significant reduction in the clinical disease activity index (Figure 2E).

**Figure 3:**
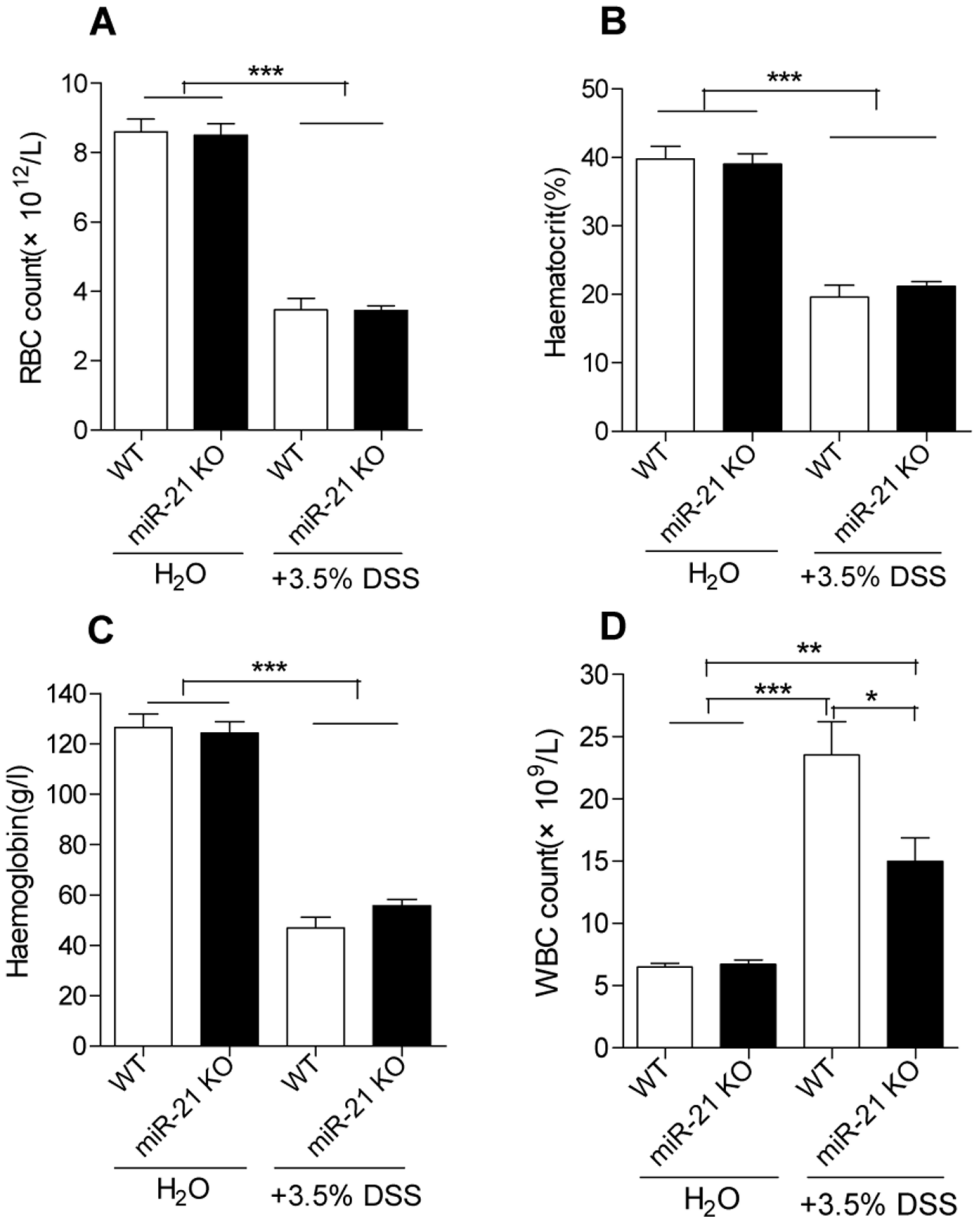
Blood parameters in miR-21 KO and WT mice following experimentally induced colitis. (A) Red blood cell (RBC) count, (B) Haematocrit, (C) Haemoglobin, and (D) White blood cell (WBC) counts in blood of miR-21 KO and WT mice were measured on 4th day of DSS administration (mean±SEM, n = 6 per group, *p<0.05, **p<0.01,***p<0.001, Student’s t test).

**Figure 4:**
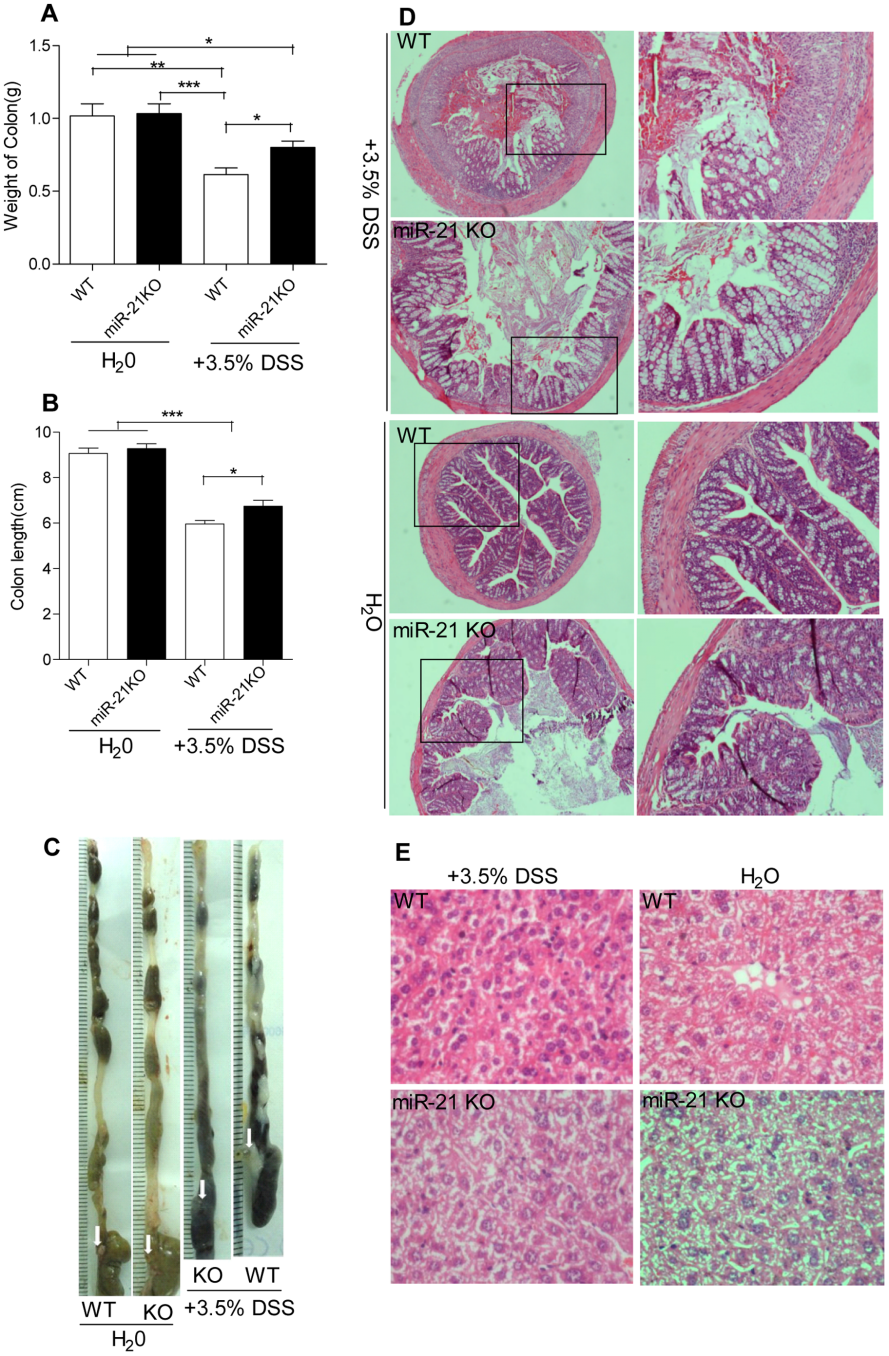
miR-21 KO mice have a attenuation in the severity of DSS-colitis. (A) Colon weight and (B) Colon length of miR-21 KO and WT mice were measured on day 7 or time of dying following DSS treatment. (mean±SEM, n≥6 per group, *p<0.05, ***p<0.001 by Student’s t test.) (C) Representative images of miR-21 KO and WT colon following of DSS or water treatment. (D) Representative photomicrographs showing epithelial damage and colonic inflammation (H&E stain, 40×magnification, higher magnification photomicrographs on the right×100) in miR-21 KO and WT mice following of DSS or water administration. (E) Representative photomicrographs showing liver damage and inflammation (H&E stain, 400×magnification) in miR-21 KO and WT mice following of DSS or water administration.

WT mice were moribund at day 5, while 100% of miR-21 KO mice survived at day 6 (Figure 2F). Furthermore, hematological parameters including white and red blood cell count, hematocrit, and hemoglobin in blood of miR-21 KO and WT mice were measured at day 4 following experimentally induced colitis. There were significant difference in red blood cells count, hematocrit, and hemoglobin between before and after DSS treatment, but no significant difference between miR-21 KO and WT mice (Figure 3A, B, C). However, there was a significantly increased white blood cell (WBC) count in WT mice when compared with miR-21 KO mice (Figure 3D). Furthermore, at day 7 of DSS administration, the colons of WT animals were significantly lighter and shorter than miR-21 KO mice (Figure 4A, B, C), and when moribund the entire colon and caecum were filled with loose, bloody stool (Figure 4C).

### Reduced Inflammatory Response in miR-21 KO Mice

**Figure 5:**
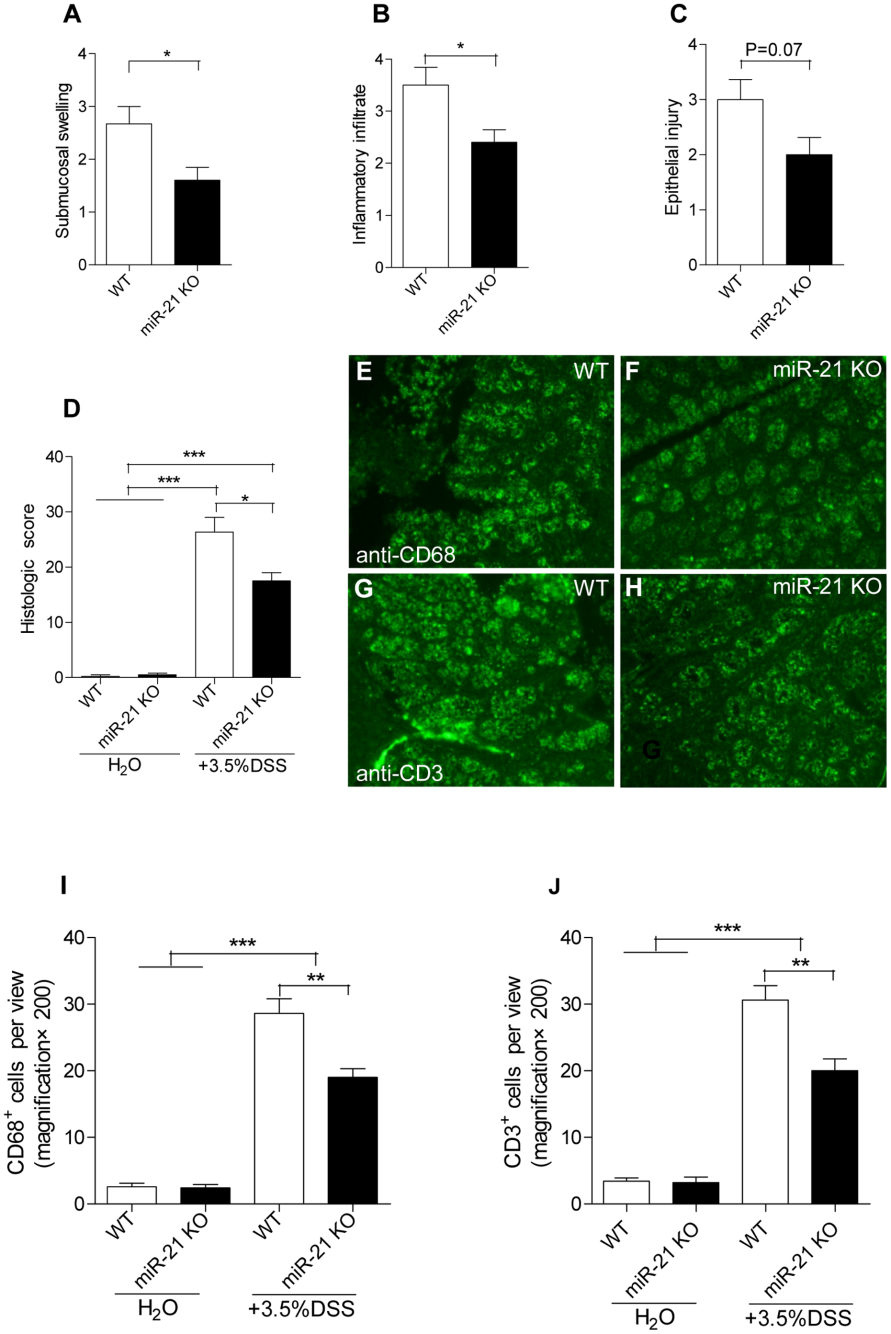
miR-21 KO mice have attenuation in intestinal epithelial injury and inflammatory infiltration. (A) Submucosal swelling, (B) Inflammatory infiltration, and (C) Epithelial injury were assessed for (D) histological colitis. (mean±SEM, n = 5 for miR-21 KO mice and n = 6 for WT mice, *p<0.05, **p<0.01,***p<0.001, Student’s t test). Colon tissue sections from miR-21 KO and WT mice were immunostained with antibodies for (E, F) CD68 and (G, H) CD3 (200×magnification). (I, J) Numbers of positive stained cells were counted per view. (mean±SEM, n = 5 per group, **p<0.01, ***p<0.001, Student’s t test).

A dramatic increase in WBC has been observed in DSS-treated WT mice when compared with miR-21 KO mice at day 4 of DSS treatment (Figure 3A). Colon architecture of miR-21 KO mice were indistinguishable from WT mice by H&E staining before DSS treatment. However, 7 days after DSS treatment, WT colon displayed more severe histopathological changes (Figure 4D). Moreover, there was more severe histopathological alteration in liver of WT mice when compared with miR-21 KO mice after DSS treatment. For instance, more cell degeneration and apoptosis and more inflammatory cell infiltration in liver can be found in WT mice (Figure 4E). At day 7 of DSS treatment, WT colons exhibited substantially greater submucosal swelling, inflammatory cell infiltration, and epithelial damage compared with miR-21 KO colon (Figure 5A, B, C). Meanwhile, WT colon displayed a significantly higher histologic score compared with miR-21 KO colon (Figure 5D). Furthermore, CD68-immunofluorescence staining detected higher numbers of monocytes in the colon sections from WT mice (Figure 5E, F, I). Similarly, CD3 positive T cells were significantly more abundant in the colon sections from WT mice when compared with miR-21 KO mice (Figure 5G, H, J).

**Figure 6:**
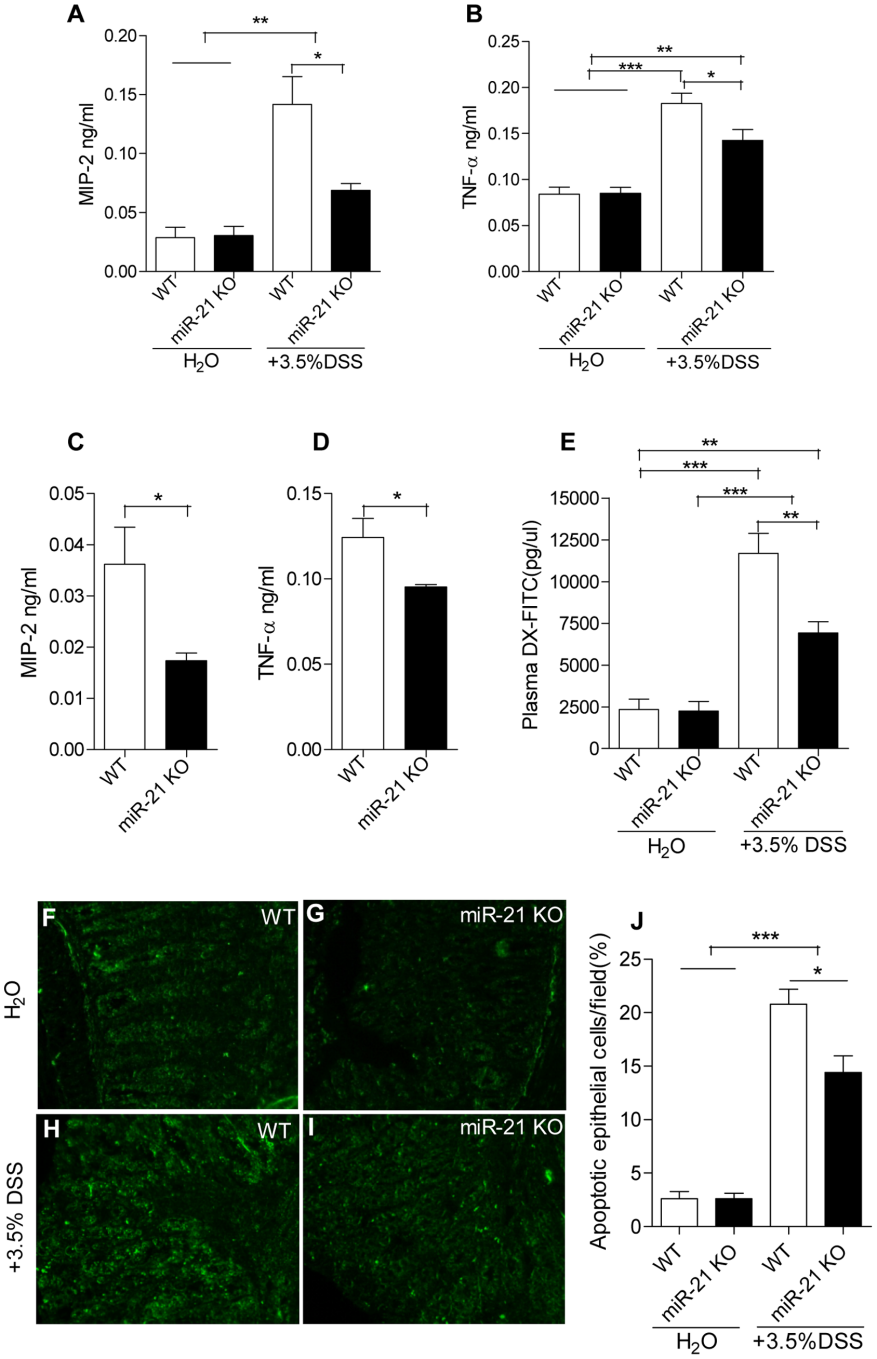
Effects of miR-21 KO on mucosal cytokine production, intestinal permeability and epithelial cells apoptosis before and after DSS treatment. Expression of macrophage inflammatory protein-2 (MIP-2) (A,C)and tumor necrosis factor α (TNF-α) (B,D) in WT and miR-21 KO mice were measured in serum (C,D) and colon culture supernatants (A,B) (mean±SEM, n = 6 per group of DSS treatment, n = 4 per group of water treatment, *p<0.05, **p<0.01,***p<0.001, Student’s t test). (E) *In vivo* intestinal permeability of WT and miR-21 KO mice before and after DSS treatment were measured by gavage with FITC-Dextran (mean±SEM, n = 5 per group, **p<0.01, ***p<0.001, Student’s t test). Fluorescence micrographs of colons in WT (F, H) and miR-21 KO (G, I) mice stained for terminal deoxynucleotidyl transferase–mediated deoxyuridine triphosphate nick-end labeling (TUNEL) (green). The *panels* are representative of 5 WT and 5 miR-21 KO mice (200×magnification). (J) Apoptotic cells were quantified by counting the apoptotic cells/field. (mean±SEM, n = 5 per group, *p<0.05, ***p<0.001, Student’s t test).

Infiltrate inflammatory activity was determined as chemokine and cytokine secretion by colonic tissue cultures. At day 7, MIP-2 and TNF-α were elevated in WT mice colon culture supernatants when compared with miR-21 KO mice (Figure 6A, B). Accordingly, MIP-2 and TNF-α were elevated in WT mice serum that obtained at day 4 of DSS treatment when compared with miR-21 KO mice (Figure 6C, D). All these experiments show that WT mice are more susceptible to colonic inflammation accompanied by near-complete epithelial erosion and extensive inflammatory cell infiltration when compared with miR-21 KO mice.

### DSS-induced Increase of Intestinal Permeability and Epithelial Cells Apoptosis were Attenuated in miR-21 KO Mice

There was no difference in intestinal permeability between WT and miR-21 KO mice before DSS treatment. The intestinal permeability was significantly increased in both WT mice and miR-21 KO mice after DSS administrated (Figure 6E). However, DSS administration leads to a more significantly increased in intestinal permeability in WT mice (Figure 6E). To investigate the consequence in cell apoptosis, we performed a TUNEL assay. After induction of colitis, WT mice displayed a significantly higher number of apoptotic epithelial cells compared with miR-21 KO mice (Figure 6H, I, J). No differences in epithelial cell apoptosis were found in miR-21 KO and WT mice before DSS treatment (Figure 6F, G, J).

**Figure 7:**
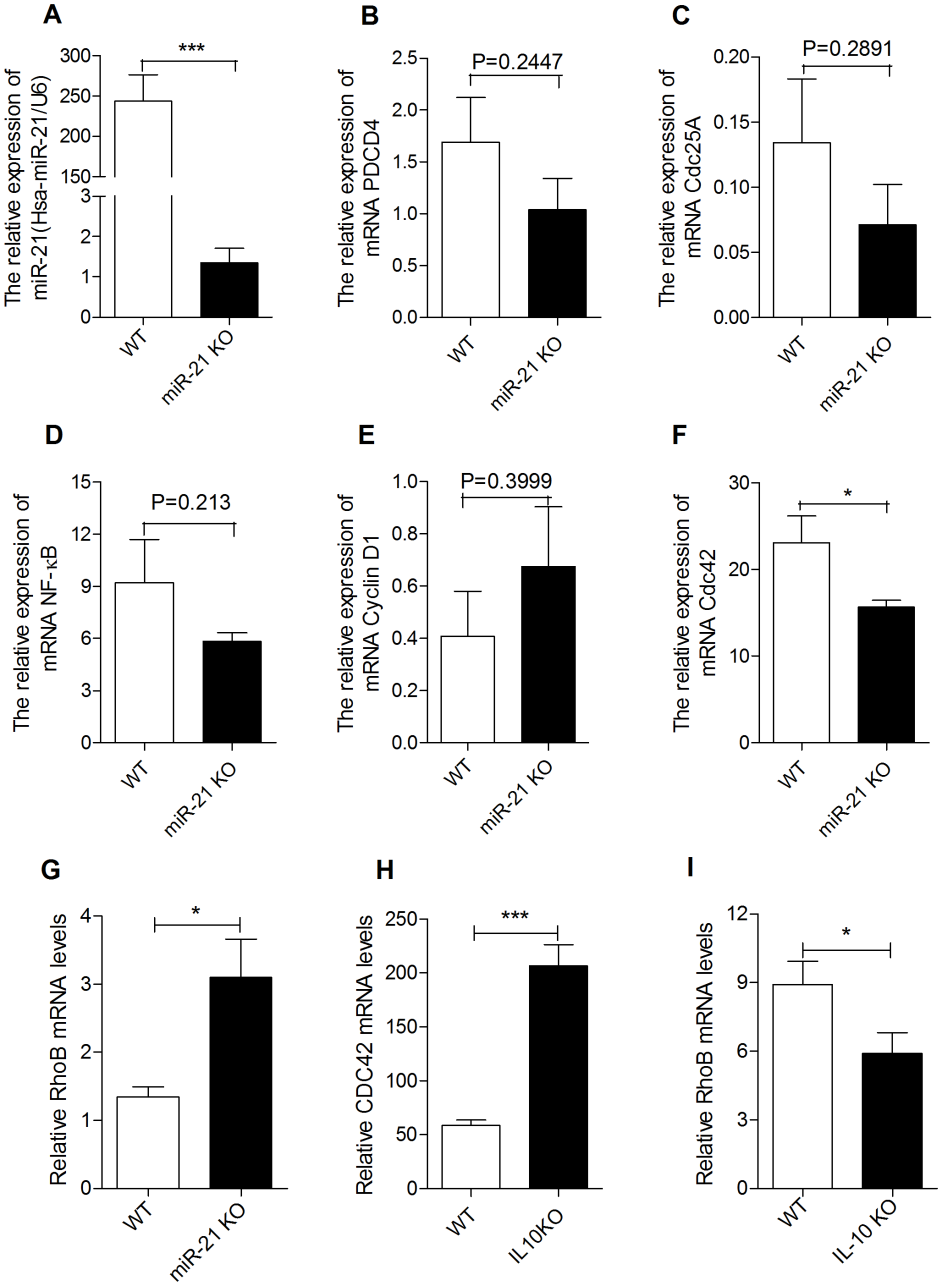
Effects of miR-21 KO on expression of relevant gene. QRT-PCR were performed to measure the expression of miR-21(A), PDCD4(B), Cdc25A(C), NF-κB(D), Cyclin D1(E), Cdc42 (F), and RhoB (G) in colon of miR-21 KO and WT mice after administration of 3.5% DSS for 7 days.(mean±SEM, n = 6 per group, *p<0.05,***p<0.001, Student’s t test). Meanwhile, we measured the expression of CDC42(H) and RhoB (I) in colon of IL-10 KO and WT mice. (mean±SEM, n = 8 per group, *p<0.05, ***p<0.001, Student’s t test).

In order to investigate the possible mechanisms that miR-21 KO attenuated DSS-induced increase of intestinal permeability and epithelial cells apoptosis, we performed QRT-PCR to investigate whether there were different expression of PDCD4, Cdc25A, NF-κB, Cyclin D1, Cdc42 and RhoB in colon of miR-21 KO and WT mice after administration of 3.5% DSS for 7 days. We verified loss of miR-21 in colon of miR-21 KO mice (Figure 7A). However, mRNA expression level of PDCD4,Cdc25A, NF-κB, and Cyclin D1 have no significant changes in colon of miR-21 KO mice(Figure 7B,C,D,E). Interestingly, Cdc42 in colon of miR-21 KO mice was significantly decreased when compared with WT mice (Figure 7F). On the contrary, RhoB was significantly increased in colon of miR-21 KO mice after DSS treatment (Figure 7G). In order to confirm these results, we measured the mRNA of CDC42 and RhoB in colon of IL-10 KO and control mice. As our prediction, in colon of IL-10 KO mice, CDC42 was significantly increased (Figure 7H) while RhoB was significantly decreased (Figure 7I). All these results suggesting that miR-21 may regulate pathogenesis of colitis through a unique molecular mechanism.

## Discussion

IBD are associated with expression changes in genes involved in immune function, wound healing, and tissue remodeling. MiR-21 acts as a potent regulator of gene expression and is differentially expressed in IBD, including UC and CD.[6]–[8] In this study, our results show that miR-21 is over-expressed in intestine of IBD and AIO patients and colon of IL-10 KO mice. Because of the similar expression pattern of miR-21 in murine experimental colitis and human IBD and AIO patients, we take advantage of miR-21-null mice to investigate its functional role in IBD pathogenesis.

Interestingly, deletion of miR-21 in mice results in a dramatic decrease in susceptibility to DSS-induced colitis, as assessed by weight loss, DAI, WBC count, histologic severity, length and weight of colon, and low mortality rates. A dramatically decreased intestinal inflammation were characterized by amelioration colitis, less leukocyte infiltration, fewer submucosal swelling, reduced epithelial damage, and weaker production of inflammatory cytokines and chemokines in the colon. In addition, we found increased level of inflammatory cytokines and chemokines such as TNF-α and MIP-2 in colon and serum of colitis mice. These cytokines could recruit inflammatory leukocytes, including granulocytes and macrophages, which could participate in tissue destruction and exacerbation of the disease. Moreover, in miR-21 KO mice, reduced colitis and injury lead to weakly histopathological alteration in liver when compared with WT mice at day 7 of DSS treatment.

All these results suggested that miR-21 may play a crucial role in regulating inflammatory and injury response in the gut. It is difficult to assess whether the up-regulation of miR-21 is a part of causative or a correlative effect in human IBD, but the experiments in mice would favor this view. What is clear is that the absence of miR-21 in the mice drastically ameliorates the development of colitis. Furthermore, miR-21 KO mice show no change in intestinal permeability and epithelial cells apoptosis in the absence of stimuli when compared with WT mice. In addition, reduced intestinal permeability and epithelial cells apoptosis in miR-21 KO mice after DSS treatment is likely due to inhibited inflammation and injury in KO mice, which lead to decreased cytokine production and epithelial cells apoptosis.

Multiple studies have suggested the central role for nuclear factor-κB (NF-κB) activation in the mucosal inflammation. [18], [34], [35] Meanwhile, miR-21 targets multiple of genes including PDCD4 and Cdc25A and modulates inflammatory processes [35] and cell cycle progression [20], respectively. Knockdown of miR-21 could decrease the level of Cyclin D1 protein and inhibit hepatocyte proliferation [19]. However, we found no significantly different expression of NF-κB, PDCD4, Cdc25A, and Cyclin D1 between WT and miR-21 KO mice after 7 days of DSS treatment. All these results demonstrate that miR-21 was not an indispensable factor to modulate target gene expression. Each miRNA may regulate hundreds of different protein-coding messenger RNA (mRNA), and conversely, a given mRNA sequence may be targeted by several miRNAs. [36] Therefore, miR-21 maybe not a key factor for induced occurs of intestinal inflammation and damage but it have an important role in modulation the severity of intestinal inflammation and damage in IBD patients. Interestingly, we found a significant difference expression of Cdc42 and RhoB between WT and miR-21 KO mice after DSS treatment. Meanwhile, we found the similar expression patterns of Cdc42 and RhoB in colon of IL-10 KO mice. Cdc42 is a master regulator of cell polarity, cell movement as well as cell cycle progression. [37], [38] In intestine, Cdc42 has been implicated in intestinal stem cell division, survival, and differentiation. [39] Furthermore, several studies have found that reduced expression or/and activity of Cdc42 was involved in reduced barrier susceptibility to infectious or inflammatory challenge.[40]-[42] Meanwhile, our latest study has found that increased miR-21 in Caco-2 cell and tissues of UC induce the degradation of RhoB mRNA, which led to the depletion of RhoB and the impairment of tight junctions in intestinal epithelial cells. [43] Thus, we speculated that miR-21 regulated pathogenesis of IBD through modulating the expression of Cdc42 and RhoB.

In conclusion, our study shows an important role of miR-21 that improve the survival rate following DSS-induced fatal colitis through protecting against inflammation and tissue injury. Furthermore, miR-21 up-regulation was observed in intestine of IBD and AIO patients and IL-10 KO mice. Therefore, down-regulated miR-21 may serve as a new approach for treatment of inflammation and injury observed in patients with IBD.
